# Early Activation of the Complement System After Brain Death in Clinical Kidney Donation and Transplantation

**DOI:** 10.1097/TP.0000000000005520

**Published:** 2025-11-19

**Authors:** Laura W.D. Knijff, Mieke F. van Essen, Sandra W. van der Kooij, Daniëlle J. van Gijlswijk-Janssen, John F. Mulvey, Maria L. Lo Faro, Rutger J. Ploeg, Cees van Kooten

**Affiliations:** 1 LUMC Transplant Center, Leiden University Medical Center, Leiden, the Netherlands.; 2 Department of Nephrology, Leiden University Medical Center, Leiden, the Netherlands.; 3 Nuffield Department of Surgical Sciences, Oxford University, Oxford, United Kingdom.

## Abstract

**Background.:**

Brain death (BD) results in an inflammatory response, including complement activation. The clinical impact of prolonged BD duration on the graft-to-be is still unclear. We investigated how BD duration impacts complement activation levels, both systemically and locally within donor kidneys.

**Methods.:**

EDTA plasma samples and kidney biopsies were obtained from the Quality in Organ Donation biobank (n = 120). Samples were routinely taken at 3 fixed points during BD management and donors were grouped according to short (≤14 h), medium (15–22 h), or long (≥23 h) duration of BD. ELISAs were used for quantification of complement in plasma, and immunohistochemistry was performed to determine complement activation at tissue level.

**Results.:**

Plasma levels of C4d, Bb, C3c, and C5b-9 were significantly elevated compared with living donor samples taken at similar timepoint. Complement activation was already observed at the start of donor management and remained elevated. Prolonged BD duration was associated with reduced complement activation, with significantly lower levels of C4d and Bb, and trends toward lower C3c. Elevated levels of Bb were associated with increased delayed graft function (DGF), while increased C4d levels showed trends toward higher DGF and lower eGFR at 3 mo posttransplantation. Also, renal biopsies taken just before reperfusion, showed local complement activation, with more intense complement staining (C3d and C5b-9) in the vascular pole in kidneys that developed DGF.

**Conclusions.:**

The complement system is already activated in BD donors early on during donor management. Prolonged BD duration was associated with reduced systemic complement activation. Increased systemic and local complement activation appears to negatively impact short-term kidney function.

## Graphical abstract

**Figure:**
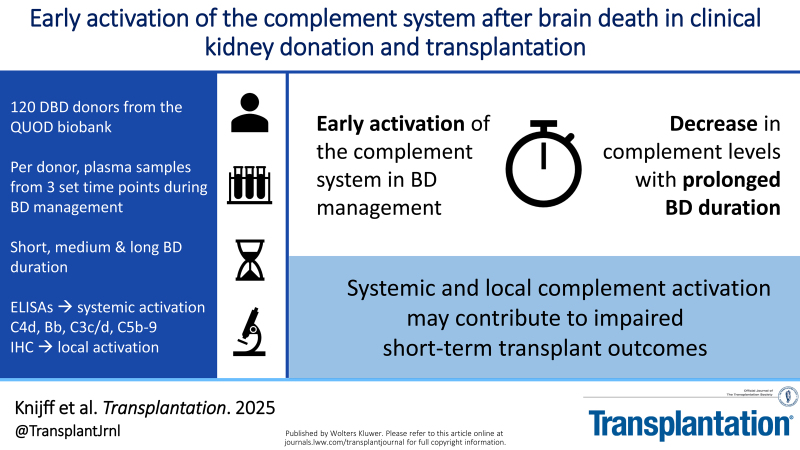

## INTRODUCTION

Donation after brain death (DBD) has long been the preferred choice of deceased donor type in solid organ transplantation. While donation after circulatory death (DCD) donors suffer from additional ischemic damage during the period of warm ischemia between the cessation of circulation and the declaration of death, DBD donor organs typically maintain oxygen and nutrient supply until procurement. Despite this advantage, organs from DBD donors with fully matched HLA profiles still result in inferior outcomes in terms of graft survival compared with organs from living donors (LDs) with HLA mismatches.^1-3^

Cerebral injury and brain death (BD) induce hemodynamic instability, leading to hypoperfusion and subsequent hypoxia in the donor kidneys, impairing their function. Expression of adhesion molecules induces leukocyte recruitment and creates an inflammatory environment in the donor kidney.^4,5^ Despite this “hostile” environment, optimized hemodynamic treatment can mitigate these effects. Also, several studies suggest that a longer duration of BD is not necessarily detrimental for transplant outcome.^6-8^ Kunzendorf et al^6^ found that kidneys from donors with a prolonged BD duration (>470 min) had an increased incidence of early graft function and improved graft survival. A validation study corroborated these findings, although no statistical significance was reached. Similarly, another study by Nijboer et al^7^ using American transplant data demonstrated slightly improved early graft function and graft survival with longer BD duration, primarily influenced by donor age. Notably, in younger donors (≤55 y), BD duration was an independent predictor of delayed graft function (DGF), while longer BD duration reduced the chance of DGF. Such beneficial effects of longer BD duration appear counterintuitive with the earlier mentioned “hostile” effects of BD on kidney function and immune activation.

The complement system is an important defense mechanism against pathogens, which comprises a protein cascade and ultimately results in the formation of C5b-9, also known as the membrane attack complex.^9,10^ Activation can occur via the classical pathway (CP), lectin pathway (LP), or alternative pathway and leads to the generation of newly cleaved complement proteins that may be used as biomarkers to measure the activation status. Damman et al^11^ investigated the role of the complement system in deceased donors and observed elevated circulating levels of C5b-9, C4d, and Bb in DBD and DCD donors compared with LDs and healthy controls. Notably, C5b-9 levels correlated with biopsy-proven acute rejection during the first year posttransplantation.^11^ However, as DBD and DCD donors were analyzed in the same cohort, specific insights regarding DBD donors and the impact of the duration of BD remain unknown.

The complement system not only plays a systemic role but also potentially has a local effect as well.^10^ Increased mRNA expression of complement proteins C2 and C3 has been found in kidney biopsies of deceased donors compared with LDs.^12,13^ In addition, complement activation has been found in renal tissue of brain-dead donors using immunohistochemistry.^10^ Also, immediately following transplantation, complement activation plays an important role. Together with reoxygenation (production of reactive oxygen species), reperfusion of the transplanted kidney with recipient blood is a trigger for complement activation and contributes to ischemia-reperfusion injury (IRI), a major contributor to graft dysfunction and rejection.^14^

While previous studies have identified either local or systemic activation of the complement system in deceased donors, to our knowledge, none have specifically studied complement dynamics during donor management following BD. A better understanding of this may help improve donor management, potentially reduce inflammatory injury in the graft-to-be and could reveal novel targets and timing for donor intervention strategies to improve transplant outcomes.

The aim of this study was to determine the activation status of the complement system in DBD kidney donors at different time points during management following the diagnosis of BD. Therefore, we examined the effects of BD duration on levels of systemic and local complement activation and assessed their association with DGF and kidney function. This study is the first to investigate complement dynamics in DBD donors, specifically in sequential samples obtained during donor management and the combination with local activation. Activation products C4d (CP or LP), Bb (alternative pathway), C3c/C3d (all pathways), and C5b-9 (terminal product of all pathways) were measured to determine the activation status of the complement system.

## MATERIALS AND METHODS

### Quality in Organ Donation Biobank

Brain-dead donors and their samples were selected from the Quality in Organ Donation (QUOD) biobank. This large national biobank routinely collects samples from most deceased donors in the United Kingdom in a standardized fashion at prespecified moments throughout the donation process.

Donor blood sample no. 2 (DB2) is collected after BD declaration, marking the start of donor management. The next sample (donor blood sample no. 3 [DB3]) is taken at the end of donor management, before the donor is moved to the operating theater for the procurement operation. The last sample (donor blood sample no. 4 [DB4]) is taken just before cross-clamping and cessation of oxygenated circulation to be followed by kidney procurement.

Blood samples are processed by centrifugation at 1300 relative centrifugal force for 15 min at room temperature and plasma aliquots are stored at –80 °C. A kidney biopsy using a core needle or punch biopsy is obtained immediately after the flushing and procurement of the donor kidney. Biopsies are stored in formalin followed by processing at the QUOD lab into formalin-fixed paraffin-embedded blocks. This study was performed under the QUOD research ethics committee approval.

### Brain Death Donor Sample Selection

BD duration was stratified into 3 groups based on the reported BD duration in deceased donors in the QUOD biobank: a first tertile of a short (≤14 h), second tertile of a medium (15–22 h), and third tertile of long (≥23 h) duration of BD. Each group included granular information of transplanted kidneys including presence or absence of DGF (defined as the need for >1 dialysis session within the first 7 d after transplantation). This resulted in a total of 6 groups with about 20 donors per group and a total of 120 donors. These 120 donors resulted in 225 kidney recipients in total.

Donors were selected based on the following criteria:

1. Availability of DB2, DB3, and DB4 EDTA plasma samples and formalin-fixed paraffin-embedded kidney biopsies from at least 50% of these donors.
2. First transplant.
3. Kidney-only transplant.
4. Available registry data on BD duration.

Groups were matched using the following variables:

1. Kidney donor risk index (UK version) based on donor age, sex, height, history of hypertension, cytomegalovirus status, estimated glomerular filtration rate at time of donation, and number of days in the hospital.
2. Total cold ischemia time.
3. Recipient variables, that is, age, weight, height, dialysis, and sex (**Table S1**, **SDC**, https://links.lww.com/TP/D304).

### Living Donor Samples

LD EDTA plasma samples were obtained from the Oxford Transplant Biobank and served as controls. Living kidney donors can be considered as “healthy” participants without exposure to a protracted duration of donor management while being brain dead but also including a procurement operation. Blood was obtained just before organ procurement, comparable to DB4, and centrifuged at 1300 relative centrifugal force for 15 min at room temperature within 2 h of collection. Plasma was stored at 4 °C and aliquoted on the same day for storage at –80 °C. Twenty LD samples were included, selected to match the samples obtained from the QUOD biobank (age: 45–63 y, sex: 50% male, height: 163–178 cm).

### Systemic Levels of C4d, Bb, C3c, and C5b-9 in EDTA Plasma Samples

Systemic complement activation was measured in EDTA plasma samples using commercial and in-house ELISAs. The following assays were performed according to the manufacturer’s instructions: C4d (MicroVue C4d EIA, A008; Quidel, San Diego, CA), Bb (MicroVue Bb Plus EIA, A027; Quidel), and C3c (HK368; Hycult Biotech, Uden, the Netherlands). C5b-9 was measured using an in-house ELISA, as described before.^15^ Samples were measured in duplicates at appropriate dilutions to be within the linear region of the standard curve. Briefly, plates were coated using a monoclonal anti-C5b-9 antibody (0.5 μg/mL, AE11, HM2167; Hycult). Samples were 15× diluted in PBS, 0.05% Tween, 1% BSA, 20 mM EDTA at pH 7.5, and detected using a monoclonal antibody against C6 (monoclonal anti-human C6 self-labeled with digoxin, A219, 1:5000; Quidel). A peroxidase-conjugated IgG fraction monoclonal mouse anti-Digoxin antibody (200-032-156, 1:10 000; Jackson ImmunoResearch, Cambridgeshire, United Kingdom) was used as the secondary antibody, and 3,3′,5,5′-tetramethylbenzidine (T4444; Sigma-Aldrich, St. Louis, MO) was used to visualize the reaction. The optical density was measured at 450 nm after stopping the reaction with 1 M H_2_SO_4_ using a VersaMax Microplate Reader (Molecular Devices, San Jose, CA) and SoftMax Pro software (version 5.4.1; Molecular Devices). Each plate included samples from all donor groups and multiple activation factors were measured on the same day to minimize freeze-thawing.

### Immunohistochemistry for C4d, C3d, and C5b-9 in Human Renal Biopsies

Sixty paraffin-embedded kidney biopsies (n = 10 per group) were obtained from the same donors as the EDTA plasma. Three sequential 4 µm slices per donor were prepared and mounted onto slides. Slides were deparaffinized and rehydrated using xylol and ethanol, while antigens were retrieved via incubation for 30 min with Proteinase XXIV (P8038, 0.1% in PBS, 1 mg/mL; Sigma-Aldrich) at room temperature. In between each step, slides were washed with PBS. Endogenous peroxidase was blocked using PBS, 0.3% H_2_O_2_, and 0.1% sodium azide for 30 min at room temperature. Slides were washed 3 times 5 min each with PBS and blocked with PBS-1% BSA for 60 min at room temperature. The primary antibodies, specific for neoepitopes on activated complement factors, were diluted in PBS-1% BSA and incubated at room temperature overnight. Slides for C4d were incubated with rabbit anti-human C4d as primary antibody (0.4 µg/mL, BI-RC4D; Biomedica, Prague, Czech Republic) and rabbit IgG (0.4 µg/mL, X0936; DAKO, Santa Clara, CA) as isotype control diluted in PBS-1% BSA. Biopsies for C3d were incubated with rabbit anti-human C3d as primary antibody (1/4000, 3.1 mg/mL, A0063; DAKO) and rabbit IgG as isotype control (0.75 μg/mL, X0936; DAKO). Both C4d and C3d are followed by goat anti-rabbit horseradish peroxidase (HRP; 1/200, 0.25 g/L, P0448; DAKO) incubation. Biopsies for C5b-9 were incubated with AE11 as primary antibody (2 μg/mL; Hycult) and mouse IgG2a as isotype control (2 μg/mL, X0943; DAKO). Goat anti-mouse HRP (1:200, 1 g/L, P0447; DAKO) was incubated for 30 min at room temperature, followed by rabbit anti-goat HRP (1:200, 2 μg/mL, P0449; DAKO) incubation for 30 min at room temperature. Deposition was visualized using NOVA Red (SK-4800; Vector Labs, Newark, CA) for 15 min at room temperature, followed by counter staining for 1 min with Mayer’s hematoxylin (Merck, 1.09249.0500). Slides were dried overnight and covered with Entellan (Merck, 1.07961) and cover slips. A 3DHistech Pannoramic 250 scanner (3DHISTECH, Budapest, Hungary) was used to scan the slides.

### Quantification of Complement Deposition Using ImageJ

Scanned slides were imaged using CaseViewer (3DHISTECH). C5b-9 deposition was analyzed based on the whole biopsy, while 5 representative ×20 magnification snapshots were analyzed for C4d and C3d. Quantification of complement deposition was performed using ImageJ software (National Institutes of Health, Bethesda, MD). The threshold of what was considered positive staining was manually determined for each biopsy but kept the same in the case of 5 snapshots. To calculate the percentage complement positive staining per area of the 5 snapshots, the total number of positive and analyzed pixels were calculated.

### Semiquantitative Assessment of Local Complement Activation in Different Kidney Compartments

Complement deposition in different kidney compartments was determined using a semiquantitative scoring table (**Figure S1**, **SDC**, https://links.lww.com/TP/D304). Biopsies were divided into: glomerulus, vascular pole, vessel, interstitial, tubule, and peritubular capillary (PTC). Each compartment was scored based on the presence and intensity of the staining with a 0, 1, or 2 score, where higher scores indicate more deposition. Scoring was performed by 2 independent researchers in a blinded manner. The average of the 2 scores was used.

### Statistical Analysis

GraphPad Prism v.9.0.1 (San Diego, CA) was used for statistical calculations and data visualization. Data are presented as the median and interquartile range. *P* ≤ 0.05 was considered significant. The Mann-Whitney *U* test was used for comparisons of 2 groups, and a Kruskal-Wallis test with Dunn’s multiple comparisons test was used to compare 3 groups. Chi-square test was used to compare differences in DGF occurrence with different systemic complement levels.

## RESULTS

### Systemic Complement Levels Are Elevated in DBD Donors Compared With Living Donor Controls

**FIGURE 1.**
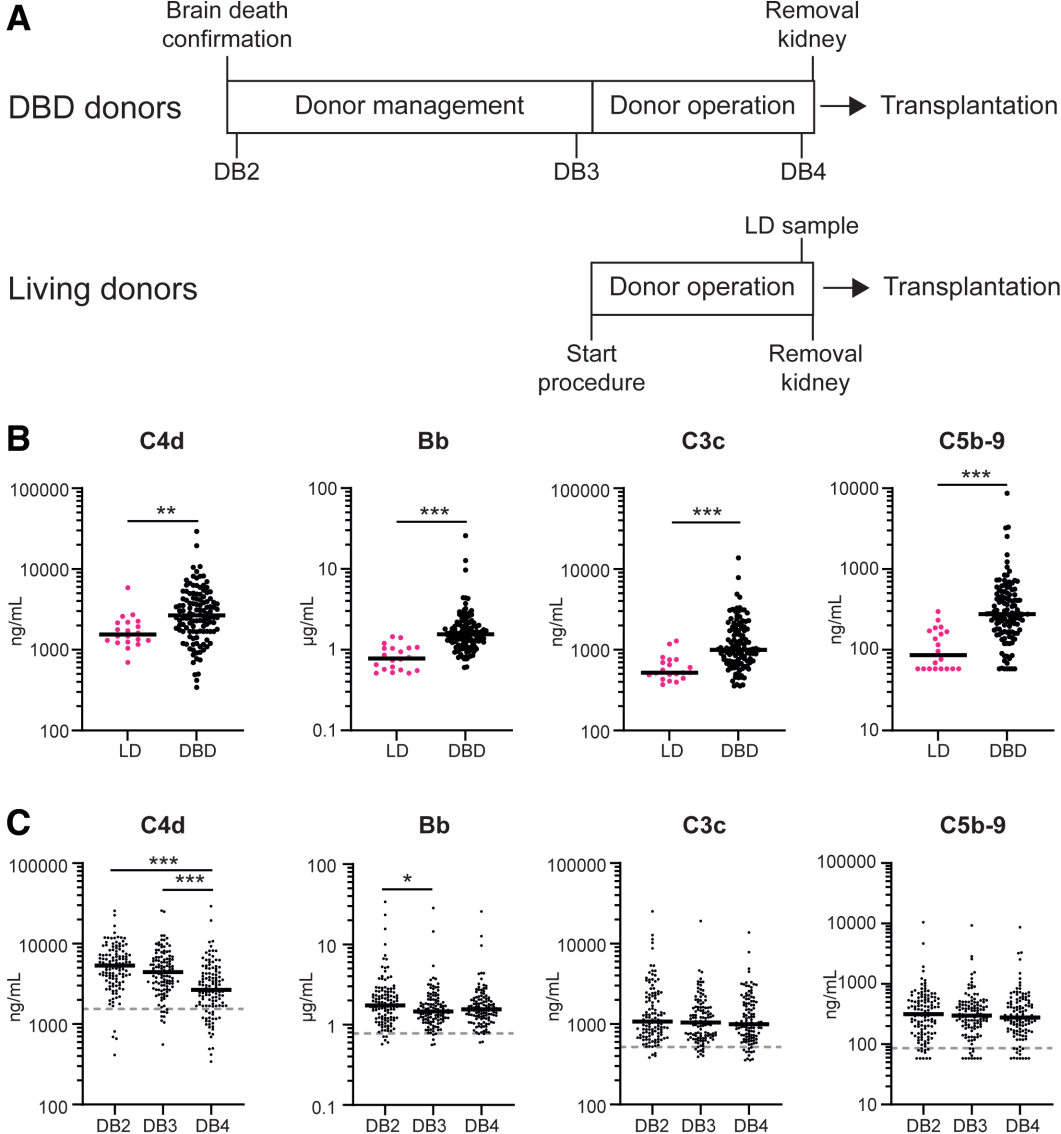
Increased complement activation in brain death donors. A, Schematic timeline of when samples are obtained from DBD donors and LDs. B, Complement activation product levels of C4d, Bb, C3c, and C5b-9 measured in EDTA plasma in LDs (n = 20) and brain death donors (n = 120). Samples from brain death donors are obtained just before organ procurement (DB4). Mann-Whitney *U* test. C, Measurement of complement activation products C4d, Bb, C3c, and C5b-9 of all DBD donors (n = 120) throughout donor management. The gray dotted line indicates the median of the levels determined in LDs, black lines indicate the median. Kruskal-Wallis test (1-way ANOVA). *P ≤ 0.05, **P = 0.002, ***P ≤ 0.001. DB2, donor blood sample no. 2; DB3, donor blood sample no. 3; DB4, donor blood sample no. 4; DBD, donation after brain death; LD, living donor.

Complement activation products were analyzed in EDTA plasma from 120 DBD donors. To investigate how these products change throughout the deceased donor management process, we investigated samples obtained at the start of donor management (DB2), at the end of donor management (DB3), and just before kidney procurement (DB4) from all these donors (schematically drawn in Figure 1A). For comparison, plasma samples were used from “healthy” LDs, obtained before procurement, at a moment similar to DB4 in the deceased donors. Before procurement (DB4), levels of C4d, Bb, C3c, and C5b-9 were all significantly increased in the DBD donors (n = 120) compared with LD (control) samples (n = 20; median C4d 2265 versus 1547 ng/mL, Bb 1.55 versus 0.78 μg/mL, C3c 996 versus 520 ng/mL, C5b-9 277 versus 86 ng/mL, respectively; Figure 1B).

Levels of complement activation products at DB4 were compared with the earlier timepoints DB2 and DB3. C3c and C5b-9 levels remained stable throughout the donor management process (Figure 1C). In contrast, levels of C4d and Bb were significantly higher at earlier timepoints (C4d median DB2 5365 ng/mL, DB3 4433 ng/mL, DB4 2664 ng/mL; Bb median 1.75 μg/mL DB2 versus 1.45 μg/mL DB3). At all timepoints, levels of C4d, Bb, C3c, and C5b-9 were found to be elevated compared with those in LDs (median, gray dotted line). Notably, there was a large variability in the levels of complement activation levels already observed at the earliest time point analyzed (DB2). To identify factors contributing to this variability, donor characteristics (sex, hypertension, cytomegalovirus status, cardiac arrest, donor type, cause of death, age, creatinine at admission, eGFR, and UK Kidney Donor Risk Index) were evaluated; however, no significant associations were found with DB2 levels (data not shown).

### Longer BD Duration Is Associated With Reduced Levels of Complement Activation

**FIGURE 2.**
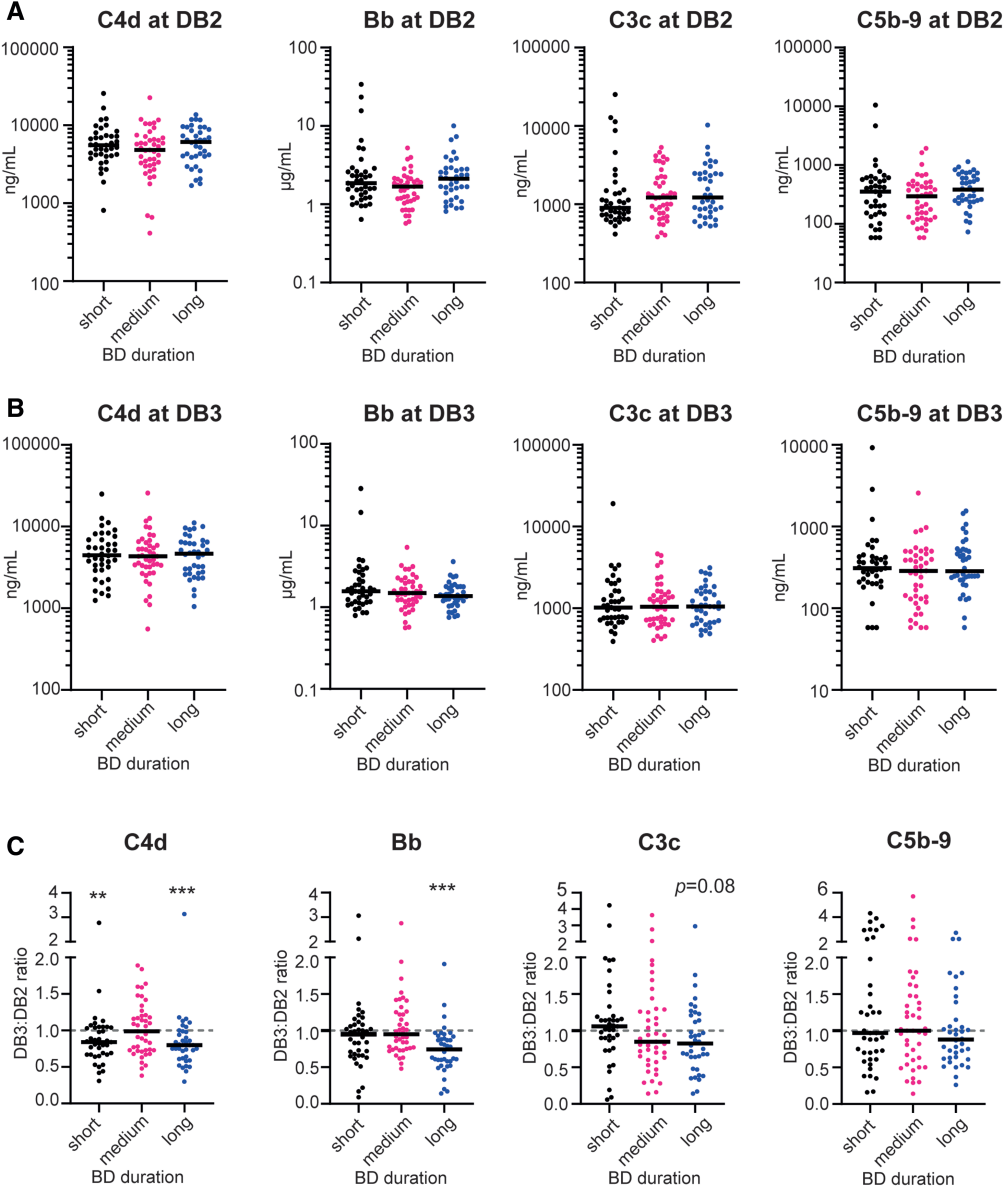
Longer BD duration is associated with reduced levels of complement activation. A, Complement activation levels at (A) DB2 (start donor management) and (B) DB3 (end of donor management) of short (≤14 h, n = 39), medium (15–22 h, n = 43), and long (≥23 h, n = 38) BD duration. Black lines indicate the median. C, Ratio of complement activation levels measured over time throughout the donor management period in DB2 (start donor management) and DB3 samples. Ratios were analyzed for decrease (>1) or increase (>1) in complement levels between DB2 and DB3. Kruskal-Wallis test. **P = 0.007, ***P < 0.001. BD, brain death; DB2, donor blood sample no. 2; DB3, donor blood sample no. 3.

To investigate the impact of the duration of BD on systemic complement activation, we excluded the DB4 samples to avoid potential additional inflammatory effects of the large and invasive donor operation and organ procurement procedure. Analysis of samples collected at the start (DB2) and end of donor management (DB3), demonstrated that levels of C4d, C3c, Bb, or C5b-9 showed no difference between a short (≤14 h), medium (15–22 h), or long (≥23 h) BD duration (Figure 2A and B).

Since we had paired samples available of all donors, we looked at individual changes in complement levels over time during BD (DB3:DB2 ratio). A significant decline in complement levels was observed for C4d and Bb in donors with a long BD duration (Figure 2C; both *P* < 0.001). Similarly, also C4d levels in the short BD duration group showed a significant decline (*P* = 0.007). Donors with a long BD duration showed a trend toward a decline in C3c levels (*P* = 0.08).

### Systemic Complement Levels May Be Associated With Reduced Short-term Kidney Function

**FIGURE 3.**
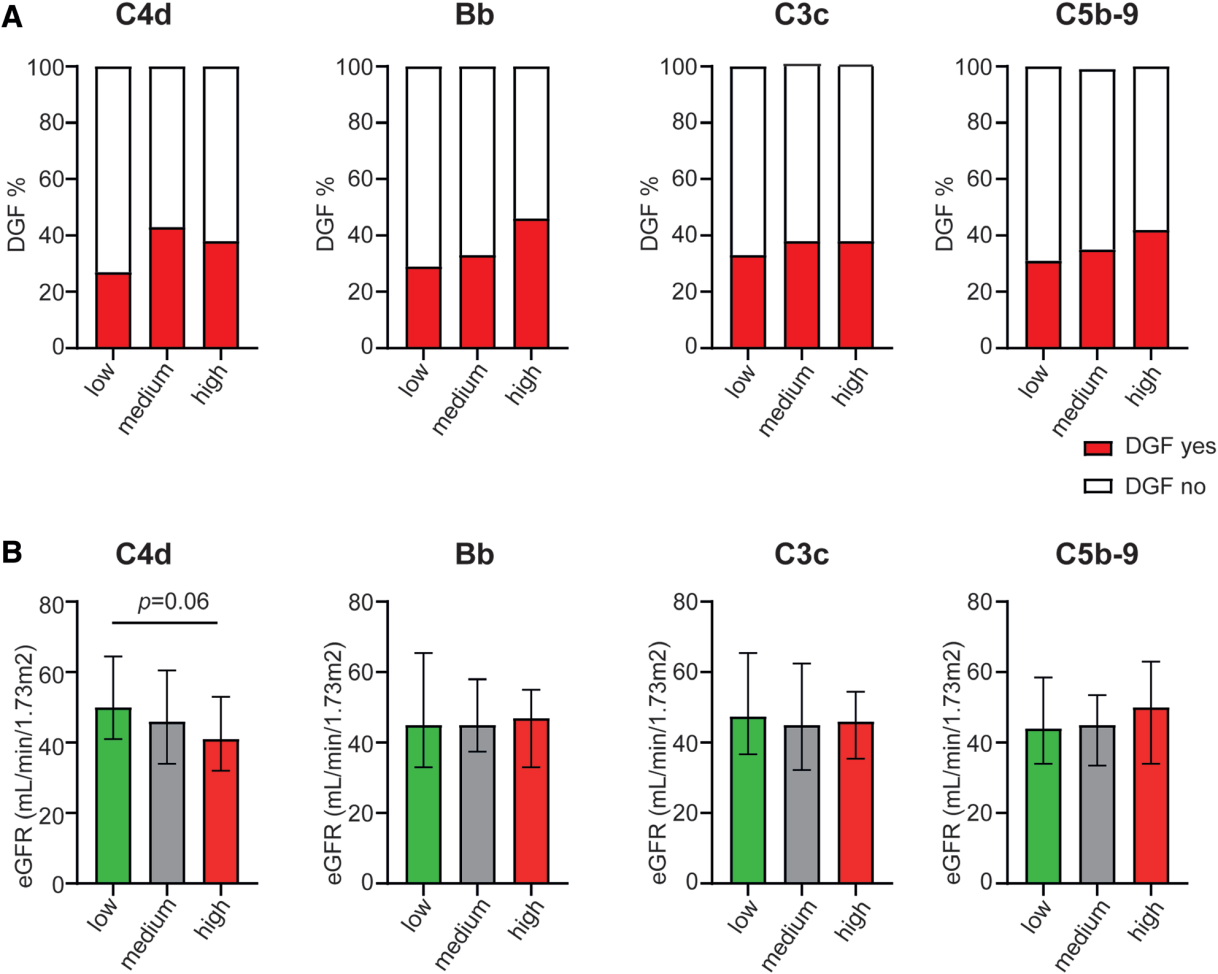
Reduced effect of complement activation levels on kidney function. A, Complement activation levels at DB4 (just before organ procurement) were divided into tertiles. All kidney recipients were included into this analysis to compare DGF outcome. C4d (low n = 71, medium n = 72, high n = 73), Bb (low n = 73, medium n = 72, high n = 71), C3c (all n = 72), and C5b-9 (low n = 72, medium n = 71, high n = 73). B, Complement activation levels at DB4 (just before organ procurement) were divided into tertiles (7 DGF status unknown and 2 primary nonfunction). All kidney recipients were included into this analysis to compare eGFR at 3 mo after transplantation. C4d (low n = 65, medium n = 65, high n = 63), Bb (low n = 65, medium n = 65, high n = 63), C3c (low n = 64, medium n = 64, high n = 65), and C5b-9 (low n = 65, medium n = 65, high n = 63; 32 recipients had missing eGFR). Chi-square test for DGF, Kruskal-Wallis test for eGFR. *P = 0.03. DB4, donor blood sample no. 4; DGF, delayed graft function; eGFR, estimated glomerular filtration rate.

Next, we investigated whether complement levels at time of organ procurement (DB4) influenced immediate- and short-term renal function. For this analysis, the transplant outcomes of both kidneys were included (when both kidneys were transplanted). Donors were grouped based on the tertiles of levels of complement activation products. Bb showed a trend (*P* = 0.07) toward a significant differences between DGF occurrence between the different Bb tertiles (Figure 3A), and was significantly higher when comparing the groups with high and low levels (29% in the low versus 46% in the high group; *P* = 0.03). When comparing the DGF occurrence in the low and high group of C4d (27% versus 38%; *P* = 0.14) and C5b-9 (31% versus 42%; *P* = 0.14) a similar trend was observed.

Short-term renal function, as indicated by eGFR at 3 mo after transplantation, showed a trend toward a lower eGFR with higher C4d levels (low median 50 versus high median 41 mL/min/1.73 m^2^; Figure 3B). The other complement activation products showed no significant differences in eGFR between the different tertiles.

To explore potential activation of specific complement pathways, levels of activation products were correlated at the time of organ procurement (DB4). A weak but significant correlation was found for C4d with C3c, but not for C4d with Bb and C5b-9. Weak but significant correlations were also found between C3c and both Bb and C5b-9, as well as between Bb and C5b-9 (**Figure S2A**, **SDC**, https://links.lww.com/TP/D304).

### Complement Deposition of C4d, C3d, and C5b-9 in Renal Biopsies

**FIGURE 4.**
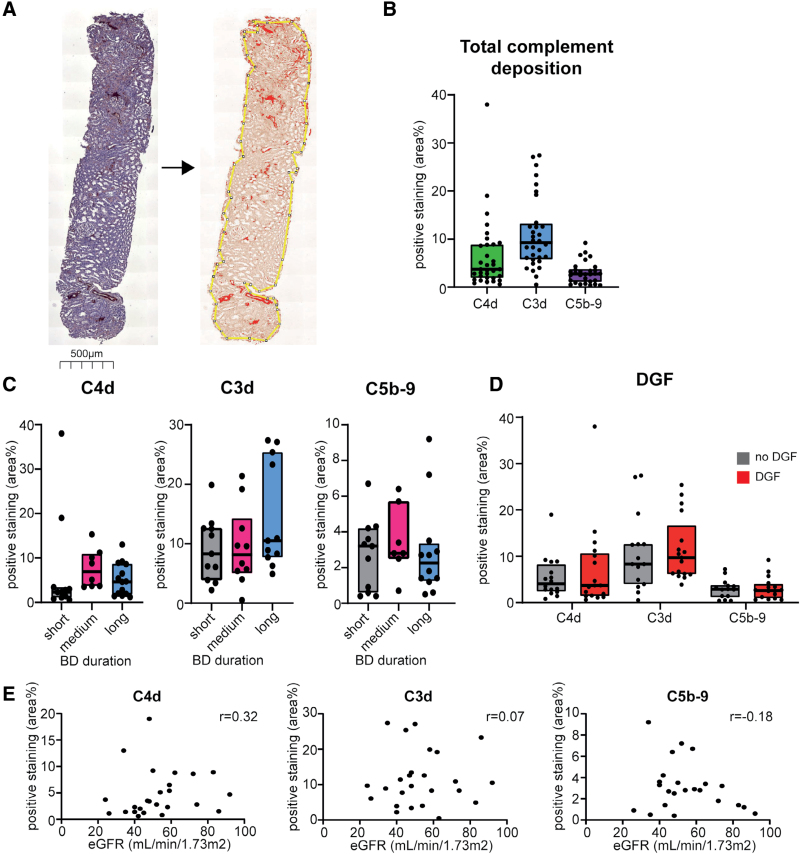
Local complement deposition is not affected by BD duration, nor does it affect subsequent kidney function. A, Representative biopsy of C5b-9 staining. Analysis with ImageJ allowed for quantification of complement deposition (positive staining per area percentage, indicated in red). B, Overview of total complement deposition of C4d (n = 33), C3d (n = 32), and C5b-9 (n = 30). C, Complement deposition of C4d (short n = 11, medium n = 8, long n = 14), C3d (short n = 11, medium n = 10, long n = 11), and C5b-9 (short n = 11, medium n = 7, long n = 12) in different BD duration groups. D, Complement deposition of C4d (no DGF n = 16, DGF n = 17), C3d (no DGF n = 15, DGF n = 17), and C5b-9 (no DGF n = 14, DGF n = 16) in kidneys that later developed DGF and those that did not. Lines and boxes indicate minimum, maximum, interquartile range, and median. E, Complement deposition of C4d (n = 25), C3d (n = 25), and C5b-9 (n = 23) in correlation with the eGFR at 3 mo after transplantation. BD, brain death; DGF, delayed graft function; eGFR, estimated glomerular filtration rate.

Kidney biopsies, obtained at time of organ procurement, were stained and analyzed for deposition of C4d, C3d, and C5b-9. C4d is a marker of CP/LP activation, whereas C3d and C5b-9 indicate potential activation of all 3 pathways. Biopsies showed a positive staining for all 3 factors, as quantified using ImageJ software (Figure 4A). The highest percentage deposition was observed for C3d (median 9.3%) followed by C4d (median, 3.7%) and C5b-9 (median, 2.8%; Figure 4B). Since the biopsies were obtained from the same donors as the plasma samples, systemic and local complement activation were compared. However, no association was observed between the 2 measurements (**Figure S2B**, **SDC**, https://links.lww.com/TP/D304), suggesting that systemic and local complement activation might be independently regulated.

To investigate whether BD duration affected local complement deposition, biopsies were stratified in the same BD duration groups as described above. No significant differences were found between short, medium, or long BD duration and positive staining (Figure 4C). Similarly, the levels of local complement deposition were not associated with DGF occurrence (Figure 4D) or the eGFR at 3 mo after transplantation (Figure 4E).

### Increased Complement Deposition in the Vascular Pole in Kidneys That Later Developed DGF

**FIGURE 5.**
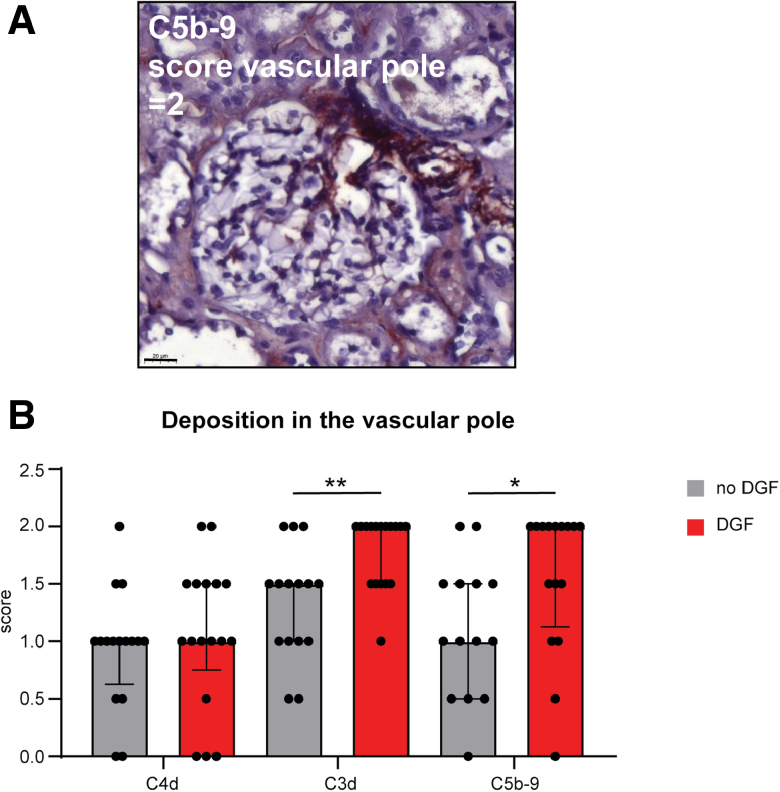
Increased complement deposition in the vascular pole in kidneys that later develop DGF. A, Representative image of staining in the vascular pole of C5b-9. Scale bar = 20 μm. B, Complement deposition of C4d (no DGF n = 16, DGF n = 17), C3d (no DGF n = 15, DGF n = 17), and C5b-9 (no DGF n = 14, DGF n = 16) in kidneys that developed DGF and those that did not. Bars show the median with interquartile range. Mann-Whitney *U* test. *P = 0.03, **P = 0.005. DGF, delayed graft function.

Since complement deposition was observed in different kidney compartments, a semiquantitative analysis was performed of the: glomerulus, vascular pole, vessel, interstitial, tubule, and PTC (**Figure S1**, **SDC**, https://links.lww.com/TP/D304). The cumulative score of the compartments correlated with the positive area score from the whole biopsy analysis from ImageJ (**Figure S3**, **SDC**, https://links.lww.com/TP/D304). C4d and C3d deposition were present in all compartments (**Table S2**, **SDC**, https://links.lww.com/TP/D304). In contrast, C5b-9 deposition was predominantly seen in the vascular pole, vessels, and PTC. The vascular pole appeared as a specific location for complement activation, as illustrated by C5b-9 deposition (Figure 5A). Further analysis showed increased deposition of C3d and C5b-9 at this location in kidneys that later developed DGF compared with kidneys with immediate function (Figure 5B; *P* = 0.005 and *P* = 0.03, respectively). No relation was found between C4d deposition in any of the kidney compartments with DGF.

## DISCUSSION

Previous studies have demonstrated activation of the complement system in deceased donors, but specific and more detailed information on DBD donors has been lacking.^10-12^ Our study is the first to assess both systemic and local complement activation at multiple time points during donor management in a well-defined DBD cohort, using high-quality EDTA plasma and biopsy samples. These DBD donor samples, collected at bespoke time points and processed in a standardized way, showed that there is increased activation of the complement system in these deceased donors compared with LD controls, already early on after BD. Importantly, we show that this early complement activation remains throughout donor management. Prolonged BD duration was associated with lower systemic complement activation levels. Besides, increased systemic activation was associated with reduced short-term kidney function as determined by incidence of DGF and eGFR at 3 mo after transplantation. We also observed that local complement deposition in the vascular pole was more prominent in kidneys that later developed DGF compared with those with immediate function.

An important observation was the fact that activation of the complement system was already present early on, at the start of donor management, with substantial variation in complement levels between donors. In healthy individuals complement levels have been demonstrated to be influenced by factors such as sex and age.^16^ Neither sex, age, hemodynamic stability, nor hypertension could explain the variation in complement levels. Similarly, it is possible that different causes of cerebral injury leading to BD can result in different levels of complement activation. Nevertheless, in this study, cause of BD could also not explain the observed variations, as intracranial hemorrhage was the predominant cause of BD in these donors. Limited data were available to determine whether the intensive care unit management played a role in the difference in complement levels. However, it should be mentioned that in the UK intensivists treat potential organ donors according to an agreed national protocol, the “optimized donor care bundle.” Future studies may focus on identifying variable(s) responsible for the observed variation in complement levels.

Upstream activation products C4d and Bb decreased with prolonged BD duration, whereas downstream C3c and C5b-9 showed a trend toward decreasing or remained relatively stable. This could possibly be explained by rapid early activation of the complement system, followed by exhaustion. Future studies should investigate levels of C4 and factor B to investigate if this is the case. Fully understanding complement dynamics is crucial for determining the optimal timing for complement inhibitor therapy.

In this study, systemic activation of the complement system in DBD donors was somewhat associated with the development of DGF and eGFR at 3 mo after transplantation. This differs from the study from Halpern et al,^17^ which did not find an association between donor complement activation levels and kidney transplantation outcomes. However, that study also included DCD donors and measured complement levels in serum samples. Since serum can lead to ex vivo activation of complement after collection of the sample,^18^ it is recommended to use EDTA plasma samples when measuring complement activation products. Besides measurements in the donor, it has been demonstrated that C5b-9 levels remain stable in recipients with immediate graft function but may increase significantly between day 0 and day 7 after transplantation in recipients with DGF.^19^ The biopsies of recipients with DGF also showed higher intensity C5b-9 staining, mainly in tubules, compared with control patients. In addition, it has been shown that elevated C5b-9 plasma levels measured 10 wk after transplantation are associated with impaired 10-y patient survival rates and lower graft survival when censored for death.^20^ Also, Damman et al^11^ demonstrated a correlation between systemic C5b-9 levels, in a combination of DBD and DCD donors, and biopsy-proven acute rejection during the first year after transplantation. In our study, no selection based on rejection was made, and our sample size was too small to validate if systemic levels correlated with rejection, which remains to be studied.

After transplantation the donor kidney is exposed to a restoration of blood flow, which leads to IRI. This process is characterized by oxidative stress, endothelial dysfunction, and immune activation, with an important role for the complement system in amplifying tissue damage.^21,22^ Activation of the complement system results in further activation of the innate and adaptive immune system, resulting in further graft injury.^21,23,24^ Our data suggest that the donor kidney is already “primed” early on during donor management for a heightened inflammatory response upon reperfusion in the recipient. Complement components such as the membrane attack complex (C5b-9) not only drive inflammation but also directly injure renal cells, exacerbating graft dysfunction.^22,24-26^ Therefore, understanding complement activation dynamics in the donor is critical, as it provides an opportunity to intervene before reperfusion injury exacerbates injury. Targeted complement inhibition—whether in the donor, during ex vivo perfusion, or immediately posttransplant—could help mitigate IRI, reduce DGF incidence, and ultimately improve graft survival.

In the past, clinical studies have shown that, despite the perception that BD creates a “hostile” inflammatory environment, prolonged BD duration is not detrimental and may even benefit kidney,^6-8,27,28^ liver,^28,29^ heart,^30^ or lung^31^ transplantation. Complement activation plays a key role in IRI, a major contributor to DGF and graft loss. Our results and others suggest that prolonged BD duration might be beneficial by reducing the inflammatory environment. This underpins the important role of an optimized donor management to maintain stability of the BD donor or even improve conditions during a prolonged management period. In this study, we focused on the donor and therefore only investigated DGF and eGFR at 3 mo after transplantation as short-term outcomes. Future research should focus on elucidating the potential impact of complement activation on (longer) posttransplant kidney function. So far, the results would not argue against the use of organs from donors with extended BD durations.

Using quantitative digital image analysis of whole biopsies, we found no significant impact of different BD durations on complement deposition or subsequent renal function. However, the number of donors where local complement activation was studied was only half that of the plasma samples, and several tissue samples had to be excluded because of technical and logistic reasons (C4d n = 27, C3d n = 28, C5b-9 n = 30 excluded), limiting our statistical power. Nevertheless, we observed increased local complement deposition of C3d and C5b-9 in the vascular pole of kidneys that developed DGF. The origin and the functional significance of this activation in the vascular pole remains unclear and needs further investigation. The juxtaglomerular apparatus, located near the vascular pole, is important for renin-release and blood pressure regulation.^32,33^ Blood pressure is important for kidney function and renin has been suggested to activate complement by cleavage of C3.^34,35^ Nevertheless, a recent study showed that renin might not have this capability.^36^ Regardless of the mechanism, our findings suggest that complement activation, both systemically and locally, may be associated with (short-term) transplant outcomes.

Although the direct impact of complement activation on long-term kidney graft function remains unclear, the complement system’s involvement highlights it as a potential therapeutic target. Our study illustrates the relevance of investigating complement activity and dynamics not only during IRI but also earlier in the donor, where complement activation is already evident. This knowledge is key for determining the optimized time to administer therapeutics, like complement inhibitors. Complement inhibition in recipients, using either anti-C5 blockade (eculizumab) or C1 inhibitor (C1INH), has been exploited clinically during the peritransplant period, with mixed results. For instance, eculizumab did not prevent the development of DGF,^37^ whereas patients who received C1INH needed fewer dialysis sessions 2–4 wk after transplantation and showed a better renal function 1 y after transplantation.^38^ A recent nonhuman primate study showed the possible beneficial effects of blocking complement activation in the donor.^39^ BD was induced in nonhuman primates who received recombinant human C1INH, which blocks the CP and LP, at 3-h intervals during 20 h of BD. After transplantation, this led to improved graft function, reduced kidney injury, and a lower incidence of DGF. However, it should be noted that C1INH not only regulates the complement system but is also able to inhibit the contact system, which could contribute to the observed effects.^40,41^

Our findings also intersect with the growing field of normothermic and hypothermic machine perfusion.^42-44^ These techniques offer opportunities to modify the graft environment ex vivo and may reduce or exacerbate complement activation depending on perfusate composition and perfusion conditions. Understanding the timing and localization of complement activation, as demonstrated in this study, will be essential to refine perfusion strategies that aim to minimize inflammatory injury before implantation.

Summarizing, this study provides new insights in the dynamics of complement activation in DBD donors. We demonstrate that the complement system is activated both systemically and locally following BD and during donor management. Strong complement activation is already observed early on during BD management. Prolonged BD duration was associated with lower systemic complement levels and increased levels of complement activation appear to correlate with short-term kidney function. Our findings highlight the importance of understanding complement dynamics and studying them earlier in the donor as well.

## ACKNOWLEDGMENTS

The authors thank Inge van der Walle for critical review of the article.

## Supplementary Material



## References

[1] Terasaki PI Cecka JM Gjertson DW. High survival rates of kidney transplants from spousal and living unrelated donors. N Engl J Med. 1995;333:333–336. 7609748 10.1056/NEJM199508103330601

[2] Gjertson DW Cecka JM. Living unrelated donor kidney transplantation. Kidney Int. 2000;58:491–499. 10916072 10.1046/j.1523-1755.2000.00195.x

[3] Floerchinger B Oberhuber R Tullius SG. Effects of brain death on organ quality and transplant outcome. Transplant Rev. 2012;26:54–59. 10.1016/j.trre.2011.10.001 22459036

[4] Koo DDH Welsh KI McLaren AJ. Cadaver versus living donor kidneys: impact of donor factors on antigen induction before transplantation. Kidney Int. 1999;56:1551–1559. 10504507 10.1046/j.1523-1755.1999.00657.x

[5] Van Der Hoeven JAB Molema G Ter Horst GJ. Relationship between duration of brain death and hemodynamic (in)stability on progressive dysfunction and increased immunologic activation of donor kidneys. Kidney Int. 2003;64:1874–1882. 14531823 10.1046/j.1523-1755.2003.00272.x

[6] Kunzendorf U Hohenstein B Oberbarnscheid M. Duration of donor brain death and its influence on kidney graft function. Am J Transplant. 2002;2:292–294. 12096794 10.1034/j.1600-6143.2002.20316.x

[7] Nijboer WN Moers C Leuvenink HGD. How important is the duration of the brain death period for the outcome in kidney transplantation? Transpl Int. 2011;24:14–20. 20819191 10.1111/j.1432-2277.2010.01150.x

[8] Ergün M Özdemir-van Brunschot DMD Donders RART. Prolonged duration of brain death was associated with better kidney allograft function and survival: a prospective cohort analysis. Ann Transplant. 2019;24:147–154. 30872563 10.12659/AOT.913869 PMC6434611

[9] Walport MJC. First of two parts. N Engl J Med. 2001;344:1058–1066. 11287977 10.1056/NEJM200104053441406

[10] Poppelaars F Seelen MA. Complement-mediated inflammation and injury in brain dead organ donors. Mol Immunol. 2017;84:77–83. 27989433 10.1016/j.molimm.2016.11.004

[11] Damman J Seelen MA Moers C. Systemic complement activation in deceased donors is associated with acute rejection after renal transplantation in the recipient. Transplantation. 2011;92:163–169. 21677599 10.1097/TP.0b013e318222c9a0

[12] Yang J Snijders MLH Haasnoot GW. Elevated intragraft expression of innate immunity and cell death-related markers is a risk factor for adverse graft outcome. Transpl Immunol. 2018;48:39–46. 29475090 10.1016/j.trim.2018.02.009

[13] Naesens M Li L Ying L. Expression of complement components differs between kidney allografts from living and deceased donors. J Am Soc Nephrol. 2009;20:1839–1851. 19443638 10.1681/ASN.2008111145 PMC2723986

[14] Danobeitia JS Djamali A Fernandez LA. The role of complement in the pathogenesis of renal ischemia-reperfusion injury and fibrosis. Fibrogenes Tissue Repair. 2014;7:16. 10.1186/1755-1536-7-16 PMC4224961 25383094

[15] De Vries DK Der Pol PV Van Anken GE. Acute but transient release of terminal complement complex after reperfusion in clinical kidney transplantation. Transplantation. 2013;95:816–820. 23348894 10.1097/TP.0b013e31827e31c9

[16] Da Costa MG Poppelaars F Van Kooten C. Age and sex-associated changes of complement activity and complement levels in a healthy Caucasian population. Front Immunol. 2018;9:411472. 10.3389/fimmu.2018.02664 PMC6255829 30515158

[17] Halpern SE Rush CK Edwards RW. Systemic complement activation in donation after brain death versus donation after circulatory death organ donors. Exp Clin Transplant. 2021;19:635–644. 33877036 10.6002/ect.2020.0425

[18] Yang S McGookey M Wang Y. Effect of blood sampling, processing, and storage on the measurement of complement activation biomarkers. Am J Clin Pathol. 2015;143:558–565. 25780008 10.1309/AJCPXPD7ZQXNTIAL

[19] Arias-Cabrales CE Riera M Pérez-Sáez MJ. Activation of final complement components after kidney transplantation as a marker of delayed graft function severity. Clin Kidney J. 2021;14:1190–1196. 33841865 10.1093/ckj/sfaa147 PMC8023215

[20] Witczak BJ Pischke SE Reisæter AV. Elevated terminal C5b-9 complement complex 10 weeks post kidney transplantation was associated with reduced long-term patient and kidney graft survival. Front Immunol. 2021;12:738927. 34759922 10.3389/fimmu.2021.738927 PMC8573334

[21] Nieuwenhuijs-Moeke GJ Pischke SE Berger SP. Ischemia and reperfusion injury in kidney transplantation: relevant mechanisms in injury and repair. J Clin Med. 2020;9:253. 31963521 10.3390/jcm9010253 PMC7019324

[22] Qi R Qin W. Role of complement system in kidney transplantation: stepping from animal models to clinical application. Front Immunol. 2022;13:811696. 35281019 10.3389/fimmu.2022.811696 PMC8913494

[23] Bongoni AK Lu B McRae JL. Complement-mediated damage to the glycocalyx plays a role in renal ischemia-reperfusion injury in mice. Transplant Direct. 2019;5:e341. 30993186 10.1097/TXD.0000000000000881 PMC6445655

[24] Zhou W Farrar CA Abe K. Predominant role for C5b-9 in renal ischemia/reperfusion injury. J Clin Invest. 2000;105:1363–1371. 10811844 10.1172/JCI8621 PMC315463

[25] Curci C Castellano G Stasi A. Endothelial-to-mesenchymal transition and renal fibrosis in ischaemia/reperfusion injury are mediated by complement anaphylatoxins and Akt pathway. Nephrol Dial Transplant. 2014;29:799–808. 24463188 10.1093/ndt/gft516

[26] Castellano G Melchiorre R Loverre A. Therapeutic targeting of classical and lectin pathways of complement protects from ischemia-reperfusion-induced renal damage. Am J Pathol. 2010;176:1648–1659. 20150432 10.2353/ajpath.2010.090276 PMC2843457

[27] Eerola V Helanterä I But A. The association of time to organ procurement on short-and long-term outcomes in kidney transplantation. Clin J Am Soc Nephrol. 2021;16:427–436. 33637606 10.2215/CJN.11420720 PMC8011019

[28] Boffa C Curnow E Martin K. The impact of duration of brain death on outcomes in abdominal organ transplantation. Transplantation. 2017;101:S1.

[29] Eerola V Helanterä I Åberg F. Timing of organ procurement from brain-dead donors associates with short- and long-term outcomes after liver transplantation. Transpl Int. 2022;35:10364. 36118016 10.3389/ti.2022.10364 PMC9472133

[30] Jawitz OK Raman V Barac YD. Impact of donor brain death duration on outcomes following heart transplantation: a UNOS registry analysis. J Thorac Cardiovasc Surg. 2020;159:1345–1353.e2. 31147170 10.1016/j.jtcvs.2019.04.060 PMC6821595

[31] Jawitz OK Raman V Barac Y. Impact of donor brain death duration on outcomes after lung transplantation. Ann Thorac Surg. 2019;108:1519–1526. 31271742 10.1016/j.athoracsur.2019.05.026 PMC6815246

[32] Briggs JP Schnermann JB. Whys and wherefores of juxtaglomerular apparatus function. Kidney Int. 1996;49:1724–1726. 8743485 10.1038/ki.1996.255

[33] Ito S. Kidney and hypertension: role of the juxtaglomerular apparatus. Tohoku J Exp Med. 1997;181:411–429. 9210249 10.1620/tjem.181.411

[34] Plasse RA Nee R Olson SW. Aliskiren as an adjunct therapy for atypical hemolytic uremic syndrome. Clin Kidney J. 2020;13:39–41. 32083614 10.1093/ckj/sfz146 PMC7025342

[35] Békássy ZD Kristoffersson AC Rebetz J. Aliskiren inhibits renin-mediated complement activation. Kidney Int. 2018;94:689–700. 29884545 10.1016/j.kint.2018.04.004

[36] Zhang Y Martin B Spies MA. Renin and renin blockade have no role in complement activity. Kidney Int. 2024;105:328–337. 38008161 10.1016/j.kint.2023.11.005 PMC10872535

[37] Schröppel B Akalin E Baweja M. Peritransplant eculizumab does not prevent delayed graft function in deceased donor kidney transplant recipients: results of two randomized controlled pilot trials. Am J Transplant. 2020;20:564–572. 31452319 10.1111/ajt.15580

[38] Jordan SC Choi J Aubert O. A phase I/II, double-blind, placebo-controlled study assessing safety and efficacy of C1 esterase inhibitor for prevention of delayed graft function in deceased donor kidney transplant recipients. Am J Transplant. 2018;18:2955–2964. 29637714 10.1111/ajt.14767

[39] Danobeitia JS Zens TJ Chlebeck PJ. Targeted donor complement blockade after brain-death prevents delayed graft function in a non-human primate model of kidney transplantation. Am J Transplant. 2020;20:1513–1526. 31922336 10.1111/ajt.15777 PMC7261643

[40] Schoenfeld AK Lahrsen E Alban S. Regulation of complement and contact system activation via C1 inhibitor potentiation and factor XIIa activity modulation by sulfated glycans—structure-activity relationships. PLoS One. 2016;11:e0165493. 27783665 10.1371/journal.pone.0165493 PMC5082678

[41] Amara U Flierl MA Rittirsch D. Molecular intercommunication between the complement and coagulation systems. J Immunol. 2010;185:5628–5636. 20870944 10.4049/jimmunol.0903678 PMC3123139

[42] Jager NM Venema LH Arykbaeva AS; PROPER study consortium. Complement is activated during normothermic machine perfusion of porcine and human discarded kidneys. Front Immunol. 2022;13:831371. 35911712 10.3389/fimmu.2022.831371 PMC9327788

[43] van Leeuwen LL Spraakman NA Brat A. Proteomic analysis of machine perfusion solution from brain dead donor kidneys reveals that elevated complement, cytoskeleton and lipid metabolism proteins are associated with 1-year outcome. Transpl Int. 2021;34:1618–1629. 34448265 10.1111/tri.13984 PMC9292651

[44] de Boer E Sokolova M Jager NM. Normothermic machine perfusion reconstitutes porcine kidney tissue metabolism but induces an inflammatory response, which is reduced by complement C5 inhibition. Transpl Int. 2024;37:13348. 39606689 10.3389/ti.2024.13348 PMC11598510

