# Supplementary material for: Early Activation of the Complement System After Brain Death in Clinical Kidney Donation and Transplantation
Source: Transplantation. 2025 Nov 19;109(12):1845–54. doi: 10.1097/TP.0000000000005520 (PMC12637106; doi:10.1097/TP.0000000000005520)
Supplement: Supplementary file 1 [file tpa-109-1845-s001.pdf]

Tables

Table S1 Donor and recipient variables. CIT = Cold ischemia time, CMV = cytomegalovirus, eGFR = estimated glomerular filtration rate, IQR = interquartile range.

**Donor variables**	**Median (IQR)**
Age	54 (45-63) years
Sex	50% male
Height	172 (163-178) cm
History of hypertension	34% yes
CMV status	49% negative
eGFR at the time of donation	97 (74-123) mL/min/1.73m^2
Days in the hospital before donation	4 (3-6)
Total CIT	763 (633-973) min
**Recipient variables**	**Median (IQR)**
Age	55 (46-63) years
Weight	82 (70-92) kg
Height	172 (164-178) cm
Dialysis	13% no
Sex	62% male

Table S2. Overview semiquantitative score. Biopsies were scored based on a scoring table (Figure S1) in a blinded manner by two independent researchers. Median and interquartile range of semiquantitative score per complement factor. N=31 for C4d, n=32 for C3d and n=30 for C5b-9.

	Glomerulus	Vascular pole	Vessel	Interstitium	Tubulus	Peritubular capillary
**C4d**	1.0 (1.0-1.0)	1.0 (1.0-1.5)	1.0 (0.5-1.0)	1.0 (1.0-1.5)	0.5 (0.5-1.0)	1.0 (0.5-1.5)
**C3d**	1.0 (0.9-1.1)	1.5 (1.5-2.0)	1.5 (1.5-1.5)	2.0 (1.5-2.0)	1.5 (0.5-1.5)	1.5 (1.0-2.0)
**C5b-9**	0 (0.0-0.5)	1.5 (1.0-2.0)	2.0 (1.6-2.0)	0 (0.0-0.5)	0 (0.0-0.0)	1.5 (1.0-1.9)

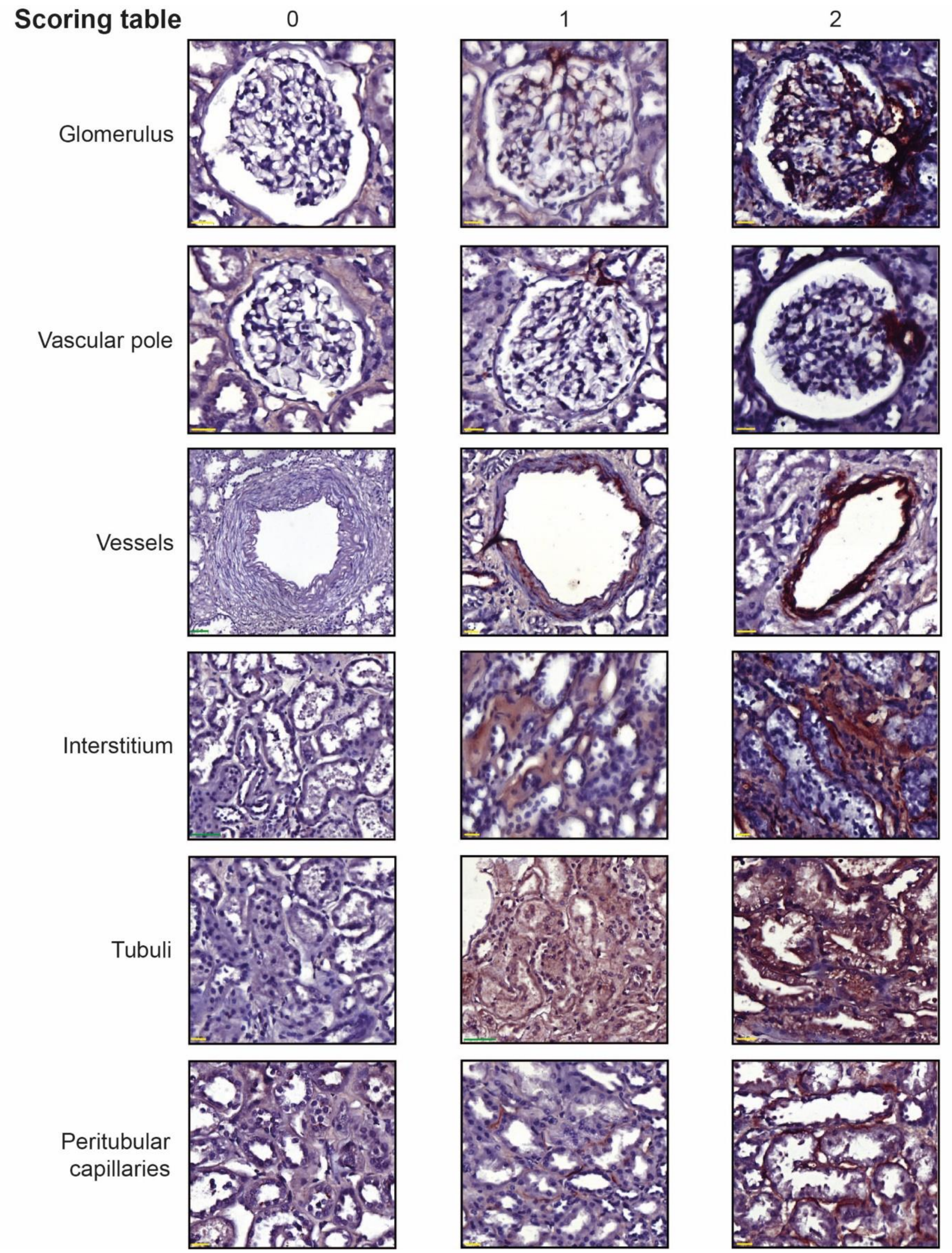

Figure S1. Scoring table for semiquantitative analysis of complement deposition. Representative images illustrate examples of scores 0 (no staining), 1 (some staining & minimum to medium intensity), and 2 (widespread & intense staining) across different kidney compartments. Images are shown for illustrative purposes only

and are derived from staining with various complement factors (C4d, C3d and C5b-9). Yellow lines indicate 20µm, green lines indicate 50µm.

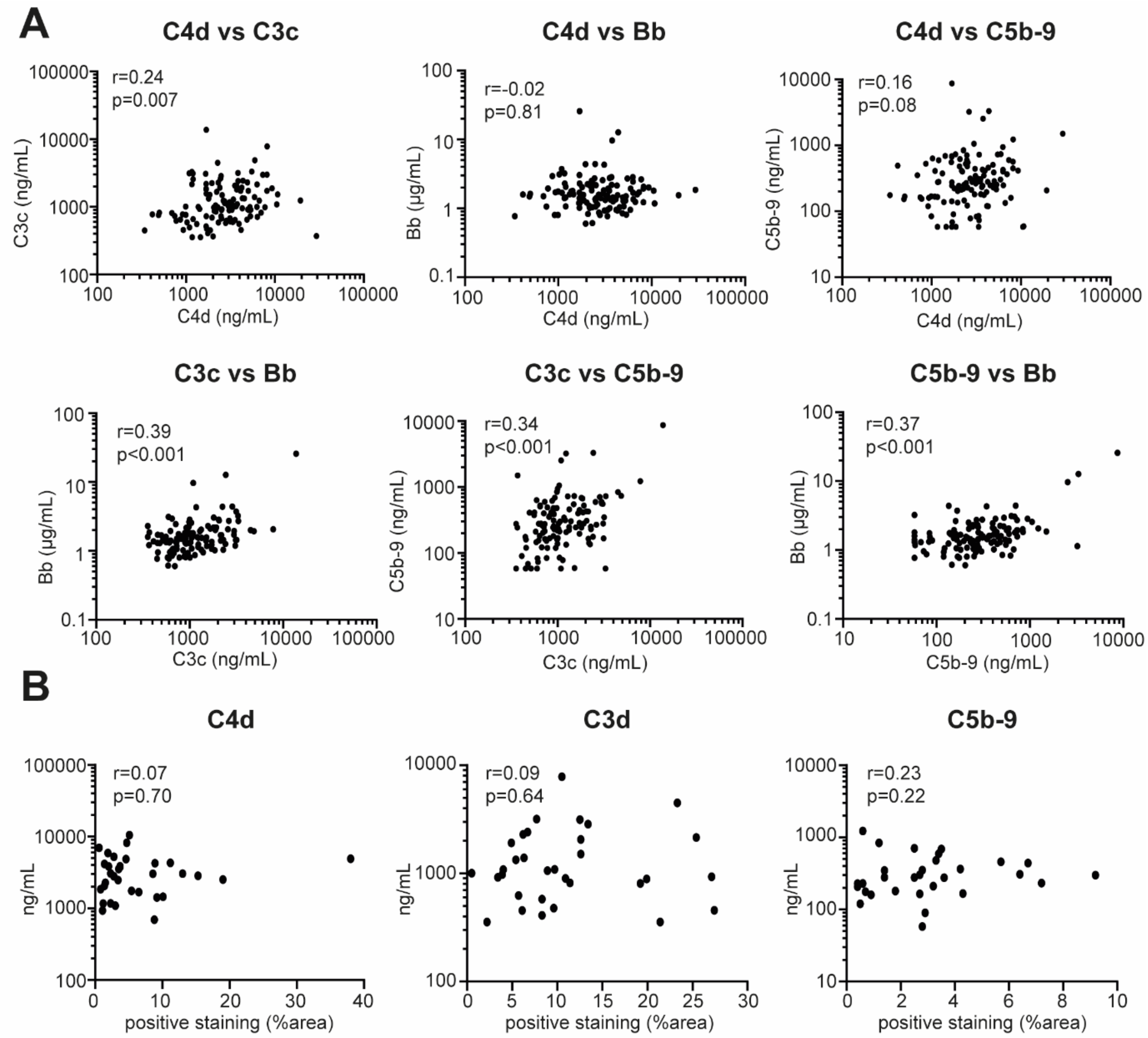

Figure S2. No correlation between complement factors on systemic and local level. A) Correlation between systemic complement levels at DB4 (just before organ procurement). B) Correlation of systemic (DB4) vs local complement levels. Spearman r test.

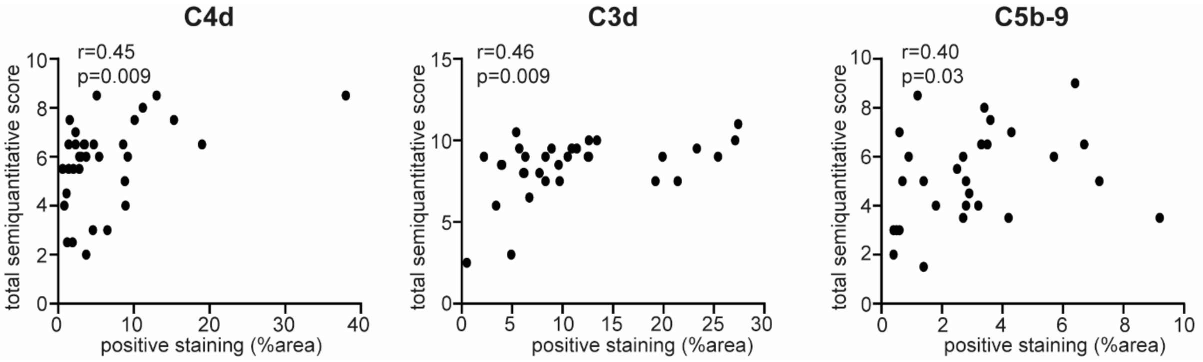

Figure S3. Correlation between quantitative and semiquantitative analysis. Correlation between quantitative analysis and semiquantitative scoring of C4d, C3d and C5b-9. Spearman r test.
